# The Weight-Inclusive versus Weight-Normative Approach to Health: Evaluating the Evidence for Prioritizing Well-Being over Weight Loss

**DOI:** 10.1155/2014/983495

**Published:** 2014-07-23

**Authors:** Tracy L. Tylka, Rachel A. Annunziato, Deb Burgard, Sigrún Daníelsdóttir, Ellen Shuman, Chad Davis, Rachel M. Calogero

**Affiliations:** ^1^Department of Psychology, The Ohio State University, Columbus, OH 43210, USA; ^2^Department of Psychology, Fordham University, Bronx, NY 10458, USA; ^3^Psychology Private Practice, Los Altos, CA 94022, USA; ^4^Directorate of Health, 101 Reykjavik, Iceland; ^5^Acoria—A Weigh Out Eating Disorder Treatment, Cincinnati, OH 45208, USA; ^6^Department of Psychology, University of Kent, Canterbury CT2 7NP, UK

## Abstract

Using an ethical lens, this review evaluates two methods of working within patient care and public health: the *weight-normative approach* (emphasis on weight and weight loss when defining health and well-being) and the *weight-inclusive approach* (emphasis on viewing health and well-being as multifaceted while directing efforts toward improving health access and reducing weight stigma). Data reveal that the weight-normative approach is not effective for most people because of high rates of weight regain and cycling from weight loss interventions, which are linked to adverse health and well-being. Its predominant focus on weight may also foster stigma in health care and society, and data show that weight stigma is also linked to adverse health and well-being. In contrast, data support a weight-inclusive approach, which is included in models such as Health at Every Size for improving physical (e.g., blood pressure), behavioral (e.g., binge eating), and psychological (e.g., depression) indices, as well as acceptability of public health messages. Therefore, the weight-inclusive approach upholds nonmaleficience and beneficience, whereas the weight-normative approach does not. We offer a theoretical framework that organizes the research included in this review and discuss how it can guide research efforts and help health professionals intervene with their patients and community.

## 1. Introduction

Jasmine is waiting in the exam room and her chart shows that her weight today is up five pounds from her last visit two years ago, putting her BMI at 32. Her blood pressure was borderline high in contrast to the normal readings in previous visits. Although Jasmine's labs were normal in past visits, they are out of date. When Dr. Johnson greets her today, Jasmine seems anxious and tells Dr. Johnson, “I almost did not come in today knowing my weight is up from the last time I was here and you suggested a diet. I feel like such a failure. However, I need help for my migraines, so here I am.” Dr. Johnson and Jasmine look at each other, there is a beat of silence, and they both sigh. Dr. Johnson thinks about all the moments like this one. Usually patients are coming in reluctantly, with medical issues that cannot wait any longer. There is a palpable sense of frustration about yet another problem related to high weight. There is a predictably tense discussion about what needs to happen. Promises are made, referrals are given, and patients drop out of sight until the next medical crisis that absolutely cannot be ignored. Dr. Johnson cannot help but think, “Could there be a better way?”

Weight management (i.e., weight loss and weight cycling) is a central component of health improvement and health care regimens in the United States and similarly westernized countries. Regardless of whether or not it is relevant to the presenting concern, patients seeking medical evaluations or treatment are typically evaluated first on the basis of their weight [1–3]. For example, primary care guidelines recommend that higher-weight individuals with a BMI above 30 should be provided with weight loss interventions and nutritional advice automatically even if their presenting concerns are unrelated to body weight [4], whereas lower-weight individuals may not be given a blood sugar evaluation because they do not fit the “high-risk profile” of a person with type II diabetes [2]. A weight-centric emphasis in medical care may overshadow patients' health concerns and needs, potentially leading to “false negatives” (i.e., failure to diagnose a true condition because a patient's weight is classified as average) in addition to the “false positives” (i.e., misdiagnosing a healthy higher-weight patient as unhealthy, thus prescribing weight loss).

The vignette above underscores the fact that many practitioners and patients are frustrated and fatigued by this process of pursuing weight loss and weight cycling [5–8], increased patient shame [9–11], and intensified weight bias from the health care provider [12–18]. Health professionals are responsible for adhering to ethical principles in the care of their patients, such as beneficience (i.e., the obligation to benefit and contribute to optimum health for patients and communities) and nonmaleficience (i.e., the obligation to avoid harming patients and communities). Yet, the dominant focus on weight loss and weight management may move health care professionals away from these principles, creating a dilemma in the delivery of ethical care and public health promotion. This dilemma occurs because a weight-normative approach to health emphasizes the pursuit of weight loss, despite extensive evidence demonstrating that weight loss is not sustainable long-term for most people [19–21] and weight cycling (commonly associated with weight loss efforts) is linked to adverse health [6–8].

In this paper, we review evidence that challenges the* weight-normative approach* for health promotion and offer evidence to support a* weight-inclusive approach* for health promotion. Instead of imagining that well-being is only possible at a specific weight, a weight-inclusive approach considers empirically supported practices that enhance people's health in patient care and public health settings regardless of where they fall on the weight spectrum [1, 2, 22]. These approaches differ in the* emphasis* each one places on weight. While health care professionals using either approach may share some commonalities (e.g., recommending similar self-care practices), they contrast in the relative importance they place on body weight in the context of health and medical treatment, their perceptions of the malleability of weight, and how they respond to patients based on their weight.

Far from being radical, we view adopting a weight-inclusive approach as more conservative than a weight-normative approach for facilitating health because it does* not* recommend a treatment option that shows more documented risks to patients than benefits. Prescribing weight loss carries the risk of adverse outcomes for adherents and lacks evidence for sustainability over time, potentially setting many patients on a path of weight cycling [23, 24]. The weight-inclusive approach acknowledges the scientific (albeit unpopular) evidence that people have little choice about what they weigh due to the interplay between involuntary genetic and environmental factors (e.g., lacking access to nutrient-dense foods priced outside of the family food budget) [25–31]. A weight-inclusive approach attempts to improve patient access to health care by recommending that health care providers recognize weight-normative biases (e.g., stereotypes that higher-weight patients must have, and lower-weight patients do not have, diseases often associated with obesity) and practices (e.g., prescribing weight-loss diets to higher-weight patients regardless of their physical health) within health care settings and challenge them in their own interactions with patients [1, 2, 32–39]. Emerging over the last four decades, this shift away from a weight-normative approach among many health care professionals acknowledges the failure of weight loss and weight management goals for improving health and recognizes the* many* factors that do support human health and well-being.

The issue of whether to adopt a weight-normative or weight-inclusive approach to health is not simply a philosophical matter. Large-scale interventions designed to affect masses of people are being implemented on the basis of the weight-normative approach. A recent scopic review of papers on the unintended harm caused by public health interventions found that over a third of the papers covered the possible harmful effects of obesity-related public health efforts [40]. Obesity-related public heath efforts were identified as potentially harmful because they (a) have been based on limited or poor quality evidence, (b) focus on preventing one extreme outcome at the expense of another extreme outcome (boomerang effects), (c) lack community engagement, and (d) ignore the root cause of the problems. If pursuit of the most ethical and effective pathways to health and well-being is the priority, and health care professionals intend to uphold the principle of doing no harm, we argue an alternative to the weight-normative approach is required. In the following sections, we review the problems and limitations of the weight-normative approach to health and then highlight the weight-inclusive approach as an alternative model for health care and health improvement.

## 2. The Weight-Normative Approach

We refer to the many principles and practices of health care and health improvement that prioritize weight as a main determinant of health as the weight-normative approach. This approach rests on the assumption that weight and disease are related in a linear fashion, with disease and weight increasing in tandem. Under the weight-normative approach, personal responsibility for “healthy lifestyle choices” and the maintenance of “healthy weights” are emphasized. On the basis of these beliefs, the weight-normative approach focuses on weight loss and weight management to prevent and treat a myriad of health problems. Despite the ubiquitous and pervasive nature of the weight-normative approach, we argue that a critical examination of the evidence does not support such a focus on weight and weight loss to improve health or prevent obesity.

First, despite the widely held belief within the medical community and general population that a higher body mass index (BMI)* causes* poor health, data do not (and cannot) support this link. The risk for mortality is highest for people with BMIs < 18.5 (underweight) and BMIs > 35 (obese II), but lowest for people with BMIs 25 to <30 (overweight), and the risk of those with BMIs 18.5 to <25 (average weight) and BMIs 30 to 35 (obese I) is comparable to and falls between the other groups [41–43]. Indeed, BMI is a corollary of certain conditions such as osteoarthritis, sleep apnea, hypertension, and coronary heart disease [44–47]. However, the data available cannot confirm that BMI causes these diseases, as causality can only be inferred via experimental designs. Other factors often partially or fully explain the links between BMI and health, such as exercise, nutrition, insulin resistance, and weight stigma [45–53].

Second, the weight-normative approach bestows negative judgments onto higher-weight individuals by promoting the view that (a) higher-weight individuals are unhealthy and thus a burden on society and (b) weight can be controlled through will power and thus if a person is fat, then it is due to poor lifestyle habits [2, 32, 54]. Given these underlying judgments, it is unsurprising that weight bias has been documented in professionals from a wide range of disciplines including physicians, nurses, psychologists, and dieticians [55]. Yet, genetic and involuntary environmental contributions to body weight outweigh voluntary lifestyle choices [25, 56–58]. Body weight is defended by a powerful biological system that reacts to a negative energy balance by lowering metabolism and increasing hunger, food preoccupation, and hedonic responses to food [26, 27]. Longitudinal research has found that children whose parents used restrictive feeding have a higher likelihood of eating in the absence of hunger and an elevated BMI later in childhood [58–60]. Lower-income families and communities may find it impossible to purchase high-quality nutrient-dense foods such as fresh fruits and vegetables given their limited budget and/or access to such foods [28–30, 61, 62]. Instead, refined grains and added sugars, fats, and preservatives are generally inexpensive and readily available in lower-income communities [28–30]. Furthermore, lower-income neighborhoods have fewer physical activity resources, such as parks, green spaces, bike paths, and recreational facilities when compared to higher income neighborhoods [63, 64]. Crime, traffic, and unsafe playground equipment are also barriers to physical activity in lower-income communities [65, 66]. Thus, there are important limitations placed on the degree to which body weight can be altered through voluntary action, making public health messages to “maintain a healthy weight” appear both uninformed and unfair.

Third, the promotion of “healthy weight” as the key to health and well-being may instill a sense of learned helplessness in the majority of people who will be unable to attain these weight-based goals [1, 2, 5, 36]. If attempts to reach and maintain a “healthy” weight continually fail or are seen as impossible given available resources, the practice of healthy behaviors may be seen as futile. Overall, there is considerable evidence that the focus on weight and weight loss is linked to diminished health. In the following sections we review a number of failures and aggravating circumstances of the weight-normative approach to elucidate why change is needed.

### 2.1. The Data behind the Failure of Weight Loss Interventions

Rising weight trends in western societies have created an intense focus on weight loss initiatives,* but none have generated long-term results for the majority of participants*. As stated by Jeffery and colleagues, despite a plethora of interventions that result in initial weight loss, participants “almost always fail to maintain the behavior changes that brought them these positive results” [20]. For example, it has been estimated that no more than 20% of participants who complete weight-based lifestyle interventions maintain weight loss one year later [21], and the percentage of people maintaining weight loss continues to drop by the second year [19]. A meta-analysis of 29 studies on structured weight loss programs conducted in the United States found that participants regained 77% of their initial weight loss, on average, after five years [67]. As it stands, these outcomes are disheartening and not encouraging, but if we actually critically evaluate these studies, it is likely that the statistics for maintenance of weight loss are even worse. That is, most of these statistics are taken from published studies and therefore may represent the most “promising” findings in terms of weight maintenance and omit data from the people who drop out and are more likely to have regained weight. Also, these studies tend to be based on rigorous trials of weight loss programs at the exclusion of more commonly employed strategies and have rigid exclusion criteria (e.g., comorbidities such as mood disorders or binge eating disorder).

### 2.2. The Data behind the Dangers of Weight Cycling

Often diet failure is accompanied by weight cycling or “yo-yo dieting”—repeated periods of weight loss and weight gain [23]. Twenty years ago, Brownell and Rodin published a foundational paper reviewing adverse medical, metabolic, and psychological health outcomes linked to weight cycling [23, 24]. Indeed, a large body of literature has connected weight cycling directly to compromised health, including higher mortality, higher risk of osteoporotic fractures and gallstone attacks, loss of muscle tissue [6–8], hypertension [51], chronic inflammation [52], and some forms of cancer such as renal cell carcinoma, endometrial cancer, and non-Hodgkin's lymphoma [50]. Here, we highlight two seminal contributions to our understanding of this link between weight cycling and compromised health. The landmark Framingham Heart Study was perhaps one of the most jarring indictments of weight cycling [6]. Using a sophisticated definition of weight cycling (capturing frequency and magnitude of fluctuations), mortality and morbidity were examined in more than 5000 individuals over a 32-year period. Results indicated that weight cycling was strongly linked to overall mortality, as well as mortality and morbidity related to coronary heart disease for both men and women. Similar results were found in the EFFORT cohort study conducted in Germany [7], which only included men, a generally underrepresented population in the weight cycling literature. In this study, 505 middle-aged men were grouped into the weight categories of stable nonobese, stable obese, weight loss, weight gain, and weight fluctuations. Among these groupings, only the weight fluctuations category was associated with mortality over the 15-year follow-up period. Of greatest interest, the stable obese category was* not *linked to higher risk of death relative to the stable nonobese category.

Weight cycling also has been shown to be connected to compromised physical health and psychological well-being. In an experimental study, Leibel et al. revealed that prospective weight loss led to reductions in metabolic energy expenditure [68]. The authors suggested that this reduction would make it difficult for their participants to maintain their newly suppressed weight. Research has shown that in order to maintain current BMI, formerly overweight dieters must eat less than their same-BMI counterparts who were never overweight [69]. As an illustration, a formerly obese woman with a BMI of 24 might be restricted to 1500 kcal/day, whereas a woman with a BMI of 24 who was never obese might be able to eat as much as 2000 kcal/day. The formerly obese woman might therefore have to employ more rigid dietary habits in order to make sure that her calories do not exceed 1500 kcal/day. Further evidence for a metabolic disruption was demonstrated in a study of 109 Korean women who participated in a community-based weight loss program [53]. Those with a history of weight cycling (43% of the sample) lost more lean muscle mass but not more body fat and lagged behind in positive changes to body composition and cholesterol, compared to their nonweight cycling counterparts, despite having lost a similar amount of weight overall.

Greater emotional distress was found to be connected to weight cycling among men and women, especially those who expected to have more personal and social success when thin (e.g., “I will be more successful, loved, desired, and healthy once I am thin/lean”), a mindset that the weight-normative approach cultivates [70]. Similarly, based on participants from the large Nurses' Health Study II, Field and colleagues found that women with a weight cycling history (39% of the sample) gained more weight over time and engaged in less physical activity but more binge eating than their noncycling peers [24]. Another recent study found that weight cycling is common among African American women (63% of the sample) and is associated with poor psychological outcomes, such as binge eating, thinness expectations, and self-esteem [71]. Overall, research conducted around the world for the past 25 years has repeatedly shown that weight cycling is inextricably linked to adverse physical health and psychological well-being.

### 2.3. The Risk of Eating Disorders in the Maintenance of Weight Loss

There is growing evidence that individuals who try to achieve and maintain a weight-suppressed state are at risk for binge eating disorder [72, 73] and bulimia nervosa [74, 75], likely because of the dietary rigidity needed to maintain a weight-suppressed state and the binge eating that may follow once the diet is “broken.” Leading researchers have found that rigid dieting is usually disrupted by episodes of overeating [72, 76] and is associated with eating in the absence of hunger [73] in experimental research. In some individuals, these temporary losses of control are accompanied by subsequent behaviors employed to compensate for calories consumed during a binge episode (e.g., vomiting, laxative misuse, excessive exercise, and fasting) [72–74]. Abrupt disruptions in caloric expenditures brought on by binge eating and the use of compensatory behaviors may also be linked to weight cycling and the associated negative outcomes described above [72, 76].

Furthermore, attempts to suppress weight are associated with poorer outcomes in treating patients with bulimia: those who have bulimia who try to maintain a weight-suppressed state are likely to binge eat [77], gain weight [75, 78], and drop out of psychotherapeutic treatment [77]. Notably, behavioral weight loss (BWL) has been considered one treatment option for binge eating disorder (BED). A rigorous examination of three approaches to treat BED found that individuals with BED who were randomly assigned to a BWL treatment group experienced a small reduction (Cohen's *d* = 0.25) in BMI after treatment but did not maintain this reduction one year or two years later [79]. The other two treatment approaches (interpersonal psychotherapy and cognitive-behavioral therapy) did not reduce participants BMI from baseline to the follow-up periods. While the other two treatment approaches reduced binge eating, BWL did not.

It stands to reason, then, that weight suppression and food restriction should not be goals of treatment. Since dieting has been associated with the onset and maintenance of eating disorders, and the cessation of dieting is a crucial step in the treatment of eating disorders, encouraging higher-weight patients to enter a weight-suppressed state by dieting is likely physically harmful and hence violates professional codes of ethics [80–84].

### 2.4. Heightened Weight Stigma under the Weight-Normative Approach

The emphasis on achieving a “healthy” weight implies that there is a healthy or normal weight that each of us should be striving to attain and maintain. Moreover, the medical endorsement of normative weights gives credibility to cultural messages prizing thinness (for women), leanness (for men), and weight loss. Internalization of socially prescribed body ideals is related to body shame, body dissatisfaction, eating disorders for women [74, 85–88], and potentially harmful muscle-enhancing and disordered eating behaviors for men [89]. The medical and cultural emphasis on “good weights” and “bad weights” produces the opportunity for weight stigma.

*Weight stigma* refers to negative weight-related attitudes and beliefs that manifest as stereotypes, rejection, prejudice, and discrimination towards individuals of higher weights [90]. There are many forms of weight stigma [91], including repeated weight-related teasing, bullying, harassment, violence, hostility, ostracism, pressures to lose weight/be thin, negative appearance commentary, and weight-related microaggressions.* Microaggressions* are intentional or unintentional verbal, behavioral, or environmental indignities that communicate hostility or negativity toward people who hold less power in society [92]. For example, suggesting a diet to a patient when the patient came in for a concern unrelated to weight would be a weight-related microaggression.* Complimentary weightism*, or appearance-related compliments (e.g., Telling a patient, “You've lost weight…looking good!”), is also stigmatizing because although seemingly positive on the surface, it still marks people as good or bad based on their weight [93].

Weight stigma occurs across a range of life domains, including school settings (higher-weight children are often stigmatized by peers, classmates, teachers, and school administrators) [94–98], health care environments (higher-weight patients are stigmatized by healthcare professionals and insurance companies) [12–15], public health initiatives [37, 99, 100], workplace settings (higher-weight employees are judged negatively by coworkers, supervisors, and employers) [101, 102], and interpersonally by loved ones (intimate partners, friends, and parents) [90, 103]. Some obstetricians and gynecologists in southern Florida have refused to perform medical services for women over 200 lbs [104]. In a large sample of women who were classified as overweight or obese, 69% experienced weight bias by a physician (with over half reporting bias on multiple occasions), 46% from nurses, 37% from dietitians, and 21% from health professionals [105]. Psychologists have been found to ascribe more pathology, greater severity of symptoms, and worse prognosis to obese patients when compared to thinner patients presenting with identical psychological profiles [106]. Weight stigma is also manifested in sociostructural barriers to accessing medical care (e.g., insurance companies that will not cover higher-weight individuals), and within the medical setting, barriers to appropriately sized equipment [3, 107]. Health care professionals' ignorance about the medical needs of higher-weight individuals, such as appropriate surgical procedures or proper dosages of medicine and chemotherapy for higher-weight individuals, is also a form of weight stigma [107].

Ironically, many professionals who treat obesity [16] and eating disorders [18] exhibit weight bias towards their patients. Health professionals who specialize in the field of obesity and weight-loss treatment demonstrate varying degrees of antifat bias, attributing negative stereotypes such as lazy, stupid, and worthless to higher-weight people [17]. Among professionals treating eating disorders, 56% observed other professionals in their field making negative comments about obese patients, 42% believed that practitioners who treat eating disorders often hold negative stereotypes about obese patients, and 35% indicated that practitioners feel uncomfortable caring for those who are obese [18]. Eating disorder professionals with stronger weight stigma were more likely to attribute obesity to behavioral causes and perceived poorer treatment outcomes for these patients. When health providers attribute weight-related stereotypes to their patients, it affects the quality of care that patients along the weight spectrum receive. Experiencing weight bias in health care settings may discourage higher-weight patients from making prohealth lifestyle changes and seeking routine or preventative care and encourage lower psychological well-being [55, 100, 105].

Due to the focus on weight evaluation and privileging thinness, even lower-weight individuals could experience weight-related stigma and microaggressions. For example, lower-weight individuals may be told that they are “hated” because they can “eat anything and still be thin,” harming their interpersonal relationships. Health care professionals may ignore lower-weight individuals' symptoms suggestive of sleep apnea and type II diabetes because they do not fit the “weight profile” tied to these conditions. Even patients who are not “flagged” for their weight may be engaging in disordered eating behaviors that are detrimental to their health (e.g., the BMI of those who have bulimia is usually in the average range [108]).

Using national survey data with a 10-year follow-up, Schafer and Ferraro found that societal weight stigma is linked to internalized weight stigma [109].* Internalized weight stigma* refers to the degree to which individuals personally adopt negative weight-based societal stereotypes and judge themselves and others based on these stereotypes [10, 110, 111]. This self-judgment may foster body blame and body shame (e.g., “If only I wasn't so large, I would not be teased—I am therefore ashamed of my body”) and appearance monitoring (e.g., vigilant about wearing slimming clothing to prevent others' from stigmatizing her body). Internalized weight bias is not related to BMI; thus, a person of any weight can experience and internalize weight bias and discrimination [112].

It is important to understand the associations between weight stigma and diminished health and well-being. Although research has challenged the assumption that high BMI causes disease, these variables do covary. One explanation for why they might covary is the experience of weight stigma [48]. Weight stigma is associated with increased caloric consumption, a pattern which challenges the common wisdom that pressures to lose weight will motivate overweight individuals to lose weight [49]. Across a 4-year longitudinal study of a large, nationally representative study of community adults, those who experienced weight stigma were 2.5 times more likely than those who were not stigmatized to become obese [113]. Priming overweight women to think about weight-related stereotypes (i.e., inducing weight stigma) led them to report significantly diminished exercise and dietary health intentions [114]. Further, Schafer and Ferraro found that weight stigma was related to increased health risks that are typically attributed to being obese, such as functional disability and decreased self-rated health, over a 10-year period [109]. The evidence further indicates that weight stigma is related to elevated ambulatory blood pressure [115], unhealthy weight control and binge eating behaviors [116–120], bulimic symptoms [121], negative body image [121–124], low self-esteem [121, 122], and depression [122, 125, 126] among children, adolescents, and adults.

## 3. The Weight-Inclusive Approach

As an alternative to the weight-normative paradigm, the* weight-inclusive approach* rests on the assumption that everybody is capable of achieving health and well-being independent of weight, given access to nonstigmatizing health care. This approach challenges the belief that a particular BMI reflects a particular set of health practices, health status, or moral character. Under this paradigm, weight is not a focal point for medical treatment or intervention. Weight is not viewed as a behavior, but eating nutritious food when hungry, ceasing to eat when full, and engaging in pleasurable (and thus more sustainable) exercise* are* self-care behaviors that can be made more accessible for people. In these ways, this approach also tries to minimize weight stigma and thus may help patients feel comfortable in the health care setting, more able to discuss their health concerns, and less likely to experience the health care encounter as stigmatizing by health care providers [3]. The weight-inclusive approach adheres to an ethical principle held by health care professions [80–84]: “above all, do no harm.” Accordingly, then, there are no set health-related interventions that prioritize BMI reduction as a goal, given that a predominant focus on BMI reduction is linked to weight stigma and internalized weight stigma, which have detrimental connections to physical health and well-being [90, 91, 100, 105, 109].

A weight-inclusive approach seeks to (a) eradicate weight-based iatrogenic practices within health care and other health-related industries and (b) end the stigmatization of health problems (i.e.,* healthism*), thereby facilitating access to health care for all individuals [1, 2, 32–39]. In taking this approach, the blame for the failure to lose weight is placed on the deleterious process of weight loss rather than on the individual, which may help minimize internalized weight stigma [32]. The weight-inclusive approach follows some general a priori principles for health professionals [1, 2, 32–39]. These principles combine in various ways and in various applications in terms of policy making, the provision of health care within practice and the community, and the patient's personal decision-making about her or his own well-being [2, 32, 39].

1. Do no harm.
2. Appreciate that bodies naturally come in a variety of shapes and sizes, and ensure optimal health and well-being is provided to everyone, regardless of their weight.
3. Given that health is multidimensional, maintain a holistic focus (i.e., examine a number of behavioral and modifiable health indices rather than a predominant focus on weight/weight loss).
4. Encourage a process-focus (rather than end-goals) for day-to-day quality of life. For example, people can notice what makes their bodies rested and energetic today and incorporate that into future behavior, but also notice if it changes; they realize that well-being is dynamic rather than fixed. They keep adjusting what they know about their changing bodies.
5. Critically evaluate the empirical evidence for weight loss treatments and incorporate sustainable, empirically supported practices into prevention and treatment efforts, calling for more research where the evidence is weak or absent.
6. Create healthful, individualized practices and environments that are sustainable (e.g., regular pleasurable exercise, regular intake of foods high in nutrients, adequate sleep and rest, adequate hydration). Where possible, work with families, schools, and communities to provide safe physical activity resources and ways to improve access to nutrient-dense foods.
7. Where possible, work to increase health access, autonomy, and social justice for all individuals along the entire weight spectrum. Trust that people move toward greater health when given access to stigma-free health care and opportunities (e.g., gyms with equipment for people of all sizes; trainers who focus on increments in strength, flexibility, V02 Max, and pleasure rather than weight and weight loss).

There are many models which include a weight-inclusive emphasis, some more fragmentary, some more comprehensive, some more focused on research evidence, some more reliant on clinical experience (while proponents lobby for new research conceptualizations and trials), and some more focused on policy and social justice while others target individual health behaviors. Such models include Health at Every Size (HAES) [1, 19, 32, 54, 127], Health in Every Respect [35], and Physical Activity at Every Size [37]. For the purposes of this paper, we explore one version in more depth, the Health at Every Size (HAES) model, as trademarked and defined by the Association for Size Diversity and Health (ASDAH) [54].

### 3.1. Health at Every Size

The HAES model comes out of discussions among healthcare workers, consumers, and activists who reject the use of weight, size, or BMI as a proxy for health and reject the myth that weight is a result of personal choices independent of uncontrollable or involuntary genetic and environmental factors [1, 19, 32, 33, 54, 127]. The HAES model addresses the broad forces that support health, such as safe and affordable access to care. It also helps people find sustainable practices that support individual and community well-being. Grounding itself in a social justice framework, the HAES model honors the healing power of social connections and evolves in response to the experiences and needs of a diverse community.

**Figure 1:**
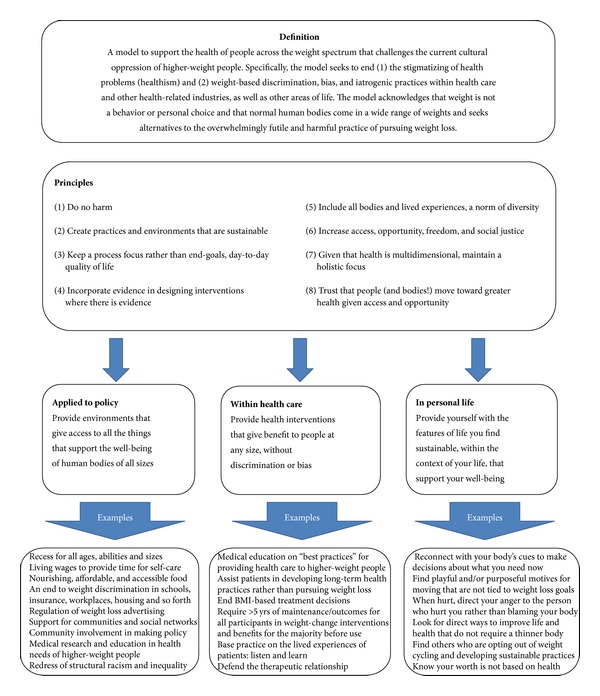
Health at Every Size (HAES): a model using a weight-inclusive approach.

The HAES model (see Figure 1) rests on the evidence that while there are links between extremes of weight and health problems,* evidence for the role of factors other than weight in people's health is stronger* [25–31]. HAES further affirms a holistic definition of health, which cannot be characterized as simply the absence of physical or mental illness, limitation, or disease, but also* the presence of quality of life* (e.g., life satisfaction), which is needed for physical health and psychological well-being [1, 32, 54]. Health should be conceived as a resource or capacity available to all regardless of health condition, ability level, or social class, and not as an outcome or objective of living. Pursuing health is neither a moral imperative nor an individual obligation, and health status should never be used to judge, oppress, or determine the value of an individual. Thereby HAES upholds the ethical principles of beneficience and nonmaleficience by focusing on eradicating weight stigma, honoring human differences (size diversity), and pursuing empirically supported interventions that promote physical health and psychological well-being (see https://www.sizediversityandhealth.org/content.asp?id=197/ for HAES Principles [32]).

Consistent with a weight-inclusive emphasis, HAES offers concrete suggestions for how to manage decisions about food and exercise in the aftermath (or absence) of a dieting mindset. HAES advocates for intuitive eating, based on evidence that demonstrates greater well-being for people who attend and respond to physiological hunger and satiety cues to determine when and how much to eat, and who pay attention to how certain foods affect the body (e.g., in terms of energy level, stamina, and medical issues such as diabetes and food allergies) [34, 128–130]. Because such individuals eat according to their internal cues the majority of the time, intuitive eating may be able to buffer situational and/or dissociative eating within environments that contain many opportunities to eat less nutritiously (e.g., fast-food restaurants, bakeries, convenience marts, etc.) [131]. Nevertheless, lack of sleep may disrupt hunger and satiety cues as it interferes with the body's leptin and ghrelin levels [132], so helping patients ensure they get adequate rest may be a goal for intervention. Years of dieting and/or the experience of clinical eating disorders may also disrupt patients' awareness of and trust in their hunger and satiety cues, and thus interventions may be needed to help patients recognize and rely on these cues [34]. HAES also argues for pleasurable movement based on evidence that exercising for pleasure in lieu of weight loss is linked to well-being and positive body image [133]. These two particular recommendations are given because people have been educated to diet and exercise for weight loss and sometimes they need concrete suggestions about how to proceed toward adaptive eating and exercise. Being compliant or rebellious about pursuing weight loss is replaced by a return to a process that honors the body's physiological signals of hunger, satiety, and need for movement.

### 3.2. The Data behind the Weight-Inclusive Approach

In addition to the data that speak against a weight-normative approach to health, there are also data in support of a weight-inclusive approach. Most of this research has focused on the HAES model and tested it against models which emphasize the weight-normative approach. Bacon and Aphramor reviewed the six existing randomized controlled trials of this research [36]. The inclusion criteria for the studies included publication in a peer-reviewed journal and an explicit focus on self-acceptance within the HAES intervention. The HAES model resulted in both statistically and clinically significant improvements for the participants on physiological measures (e.g., blood pressure), health practices (e.g., increased physical activity), and psychological measures (e.g., self-esteem and disordered eating). HAES achieved these health improvements more successfully than models that emphasize dieting. The participants within the HAES groups also demonstrated increased adherence (reduced dropout rates) and no adverse outcomes [36].

To take one illustrative example, a HAES-based program that emphasized intuitive eating and size acceptance was evaluated against a dieting-based weight-loss program with a sample of 30- to 45-year-old women classified as overweight or obese [19, 127]. Participants within each program received six months of weekly group interventions followed by six months of monthly aftercare group support. Findings yielded more positive results for the HAES-based program over the 1-year [127] and 2-year [19] follow-ups. Specifically, the HAES group decreased total cholesterol, low-density lipoprotein (LDL cholesterol), triglycerides, and systolic blood pressure at the 2-year follow-up and sustained improvements from the 1-year to 2-year follow-ups. Whereas the dieting group lost weight and showed initial improvements on many variables at the 1-year follow up, they had regained weight and did not sustain improvement at the 2-year follow- up [19]. The HAES group decreased eating restraint, physical hunger rating, disinhibited eating, drive for thinness, bulimic symptomatology, body dissatisfaction, poor interoceptive awareness, depression, and body image avoidance and increased self-esteem at both 1-year and 2-year follow-up. Correspondingly, participants in the dieting-based program only reduced disinhibited eating but reported* decreased *self-esteem [19]. Furthermore, attrition was higher in the diet group (41%) compared to the HAES group (8%) [19, 127]. These findings suggest that HAES-based interventions demonstrate better adherence to practices that promote physical health and psychological well-being than dieting-based interventions, and these effects can be sustained over time.

The focus on weight loss in the weight-normative approach could be understood by patients as promoting thinness as a goal, whereas the idealization of thinness (i.e., thin-ideal internalization) and pursuit of thinness are challenged within the weight-inclusive approach. Research on secondary eating disorder prevention efforts has also provided evidence in support of the weight-inclusive approach. For example, in their program of research on the* Body Project*, Stice and Presnell examined whether reducing participants' thin-ideal internalization and focus on weight loss would reduce their dysfunctional eating attitudes and behaviors [134]. In this program, participants engaged in a series of verbal, written, and behavioral exercises in which they actively critiqued the thin ideal. These exercises were intended to produce cognitive dissonance, such that their original attitudes (e.g., “I want to be thin,” “only if I am thin will I be beautiful”) would conflict with their recent behavior (e.g., role playing where they convince other girls that many body types are beautiful). To decrease their cognitive dissonance, participants changed their original prothinness and proweight loss attitudes to make them fit with their recent behavior of rejecting the thin ideal. Overall, the Body Project has been effective in helping early-to-late adolescent girls reduce their pursuit of the thin ideal, accept their bodies, improve mood, decrease eating disorder symptoms (e.g., binge eating and use of unhealthy weight control behaviors), and lower the risk for developing future symptoms [135].

### 3.3. Reducing Weight Stigma under the Weight-Inclusive Approach: A Model and Strategies

**Figure 2:**
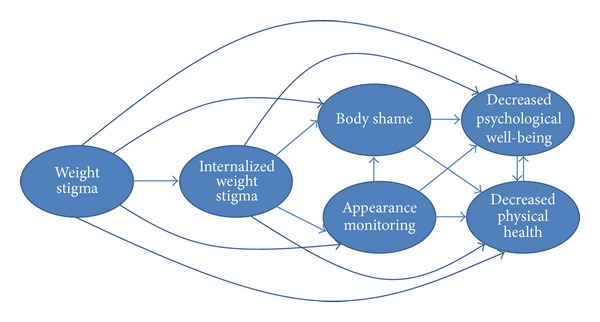
Theoretical model of weight stigma and its associated variables.

Health care professionals need to work to reduce cultural and interpersonal weight stigma within health care and their patients' environments in order to facilitate the processes that bolster physical health and psychological well-being. On the basis of the evidence for the links between weight stigma and adverse health and well-being reviewed previously [109, 113–121], and the intervening variables that could help explain these links, we devised a theoretical model (see Figure 2) that organizes these variables and the associations between them. This model can be used to help health care professionals identify points of intervention to reduce weight stigma and the other model variables that may maintain lower physical health and well-being.

Similar to other theoretical models that positioned sociocultural influences as the source for negative body image and dysfunctional self-care behaviors [136–138], we positioned weight stigma as the starting point for negative health. In light of weight stigma's associations with internalized weight stigma [109], lower physical health [109, 113–115], lower psychological well-being [116–121], body blame and shame [121–124], and appearance monitoring [130, 139], proponents of the weight-inclusive approach challenge health care providers to examine their own biases around weight. These biases are part of a wider cultural climate of weight stigma that pervades health care education and everyday life. It is possible that much of the healing power of the health care relationship is social—in the quality of the connection between health care providers and their patients and their mutual trust and regard [140]. This connection is threatened for patients by the experience of being stereotyped and reduced to a BMI category. Quality of care for higher-weight patients can be optimized by adopting effective and sensitive strategies to communicate with all patients along the weight continuum [55, 100]. Given the enormous social pressures to focus on weight loss and to connect weight loss to health, we know that providers, even those with the best of intentions, may unintentionally give the impression that they are biased against higher-weight patients, leaving their patients feeling unwelcome, invisible, and shamed.

One way health care professionals could engage with higher-weight patients is to view their office environment through a weight-inclusive lens. Does the office set-up communicate to* all* patients that their healthcare needs will be met there without shame or discrimination? Or is the office stigmatizing from the moment they arrive? For example, do waiting and exam rooms have furniture that fits higher-weight individuals? Do office staff automatically weigh in every patient, on a scale in a public hallway, even if the patient is coming in for an issue totally unrelated to weight, for example, a wart removal? How do nurses respond when a patient says, “no thank you” to being weighed? What is the office culture around weight? Has weight bias ever been addressed by the entire staff, such as through continuing education or sensitivity training? Are gowns and medical equipment (e.g., blood pressure cuffs) stocked to fit higher-weight patients?

By being a source of support and “grounding” against the stigma higher-weight patients regularly face, the weight-inclusive approach may facilitate patient adherence to health promoting practices and the guidance of their health care providers. Health care professionals can offer this support through the provider-patient bond and by connecting individuals via support groups (in person or online) that follow the weight-inclusive approach. For example, HAES has a website that could be useful to recommend for patients (http://www.haescommunity.org/).  Table 1 provides a list of weight-inclusive principles and examples of how health care providers can implement them in practice. We recognize that various health care professionals need to work as a team to fully implement these principles, with each professional implementing the principles within her or his boundaries of expertise.

In addition to the above strategies, a weight-inclusive approach includes a focus on intrapersonal variables that sustain poor physical health and well-being (see Figure 2). For instance, health care professionals can become educated about the links between internalized weight stigma and poor self-care that maintain adverse physical health and negative psychological well-being [9, 110–112, 121, 127], for example, and share this knowledge with their patients. Health care professionals can also inform patients of the rich literature that explicates the bidirectional influences between physical health and well-being [141–145]. That is, if patients begin self-care practices that enhance physical health, they will likely* feel better* psychologically as well, and these psychological gains are then linked to further increases in self-care practices that enhance health.

Health care professionals can also target internalized weight stigma's links with body shame and appearance monitoring (see Figure 2). In particular, patients often blame and shame their bodies for how they look and feel, but body blame and shame are often responses to the wider cultural stigma around weight and their personal experiences of weight discrimination over their lifetimes [121–124]. Body blame and shame can be reframed for patients to communicate that the source is likely internalized societal weight stigma [112, 146] from being stigmatized for their weight [109], and not their bodies' actual weight or size [147]. Health care professionals can also help patients mentally shift from habitual appearance monitoring, which is associated with lower self-care and ignoring physical health [85, 148, 149], to attending to their bodies in more positive ways that emphasize self-care. There are some interventions (e.g., self-compassion) that can enhance patients' well-being in tandem with improvements in body shame [150]. The key is for both health care professionals and patients to appreciate the extent to which body loathing and shame is associated with* reduced *engagement in self-care [85, 120]. There is a cultural belief that people have to be dissatisfied with their weight (or any aspect of their appearance) in order to be motivated to improve it. This belief has not found general support in the literature; in fact, the reverse is supported: people are more likely to take care of their bodies when they appreciate and hold positive feelings toward their bodies [133, 147, 151].

In order to encourage self-care behaviors, patients also need to* reconnect* with their bodies, that is, focus on internal body awareness rather than engage in external appearance monitoring [85, 147, 148, 151] (see Figure 2). Internal body awareness is required to be able to know when something is “not right” with their bodies as well as attend to their bodies' physical and psychological needs [147]. For example, awareness of hunger and satiety cues is needed to determine when and how much to eat in order to prevent under- or overeating [148, 151]. Raising internal bodily awareness could be facilitated by offering unconditional acceptance of people's bodies and bodily experience in lieu of weight stigmatization. Indeed, women who received body acceptance by others (in contrast to weight stigma) reported higher body appreciation and less habitual appearance monitoring [148, 151]; thus, they are more connected to the functionality of their bodies and less shameful of their bodies. Moreover, body acceptance by others fully accounted for the link between women's BMI and their body appreciation [151]. This finding underscores the need to eradicate all health care interventions that foster weight stigma to improve patients' perceptions that the health care environment and health care professionals accept their bodies. Greater internal awareness and appreciating the body are related to higher eating based on physiological hunger and satiety cues and less situational and emotional eating [148, 151]—additional reasons for health care professionals to encourage clients to appreciate their bodies and listen to their bodies' internal cues.

### 3.4. A Weight-Inclusive Approach to Public Health

The current public health model operates through the identification of risk factors and population-based efforts to reduce such risks in order to prevent disease and promote health [99]. The reduction of risk factors occurs through various forms of public action, including regulatory efforts (e.g., taxing and legislation), community-based universal programs (e.g., Health Promoting Schools), and public health messaging to raise awareness of the risks and benefits associated with certain behaviors (e.g., “*5-a-day*”). However, this model has been criticized for focusing too heavily on factors that are perceived to be under personal control while neglecting the larger sociocultural and economic conditions that dictate much of people's lived experiences, choices, and opportunities [37, 99].

Syme pointed out that the conditions often referred to as “lifestyle diseases,” under which overweight and obesity are named, have been associated with a variety of genetic and environmental factors that occur well outside personal control [99]. This lack of control is especially true for populations that face most health challenges. Populations with the worst health outcomes tend to also be populations living under the most socioeconomic constraints and have the least amount of personal control over their lives [31]. Marmot has written extensively about the contributions of social and economic inequalities to public health issues and the critical importance of considering these issues in public health policy [152]. Disregard for the environment within which people live offers “a rather decontextualized” approach to public health that is unlikely to be effective and may even be unethical due to the potential for harm [37].

An approach to public health that incorporates a weight-inclusive approach may not only circumvent the adverse health and well-being consequences linked to the weight-normative approach but also may enhance population health. Longitudinal studies have repeatedly shown that, irrespective of actual weight, body satisfaction and freedom from weight-based teasing and stigma are linked to reduced risk for unhealthy dieting practices, sedentary behaviors, eating disturbances, and weight gain among young people [95, 153, 154]. Public health messages that are free of weight focus also appear to be more acceptable to the public and more likely to encourage healthy behaviors than messages emphasizing weight control or obesity prevention. For example, a large nationally representative U.S. survey revealed that participants responded most favorably to public health messages that promoted healthy behaviors without any reference to weight or obesity* at all* [155]. The survey further showed that messages perceived as weight stigmatizing were negatively received and rated less likely to foster healthy behavior change. The findings have since replicated in randomized controlled settings [156].

Several scholars have proposed actions that may be taken at the policy level to prevent and reduce harm associated with a weight-focused sociocultural climate [35, 157, 158]. However, a serious, inbuilt resistance to change appears to be present within health systems. For instance, OReilly and Sixsmith have argued that an overreliance on the dominant position of powerful institutions, such as the World Health Organization, has resulted in a dead-lock situation where public health authorities uncritically accept and maintain the weight-normative approach without scrutinizing its validity, effectiveness, or ethical implications [158]. Thus, the weight-normative approach becomes a self-perpetuated dogma. The indications of harm associated with this paradigm, however, demand that a closer look be taken and actions to reduce the focus on weight within public health be implemented. Certainly, during this implementation phase, data would be needed to evaluate the outcomes of moving away from a weight-normative toward a weight-inclusive approach.

OReilly and Sixsmith analyzed policy options that could be used to shift the weight-normative approach to a more weight-inclusive approach in public health [158]. They conducted interviews with key stakeholders who were asked to rank proposed policy changes in terms of estimated effectiveness in challenging the weight-normative approach, likelihood of promoting equity and reducing weight bias, political and public acceptability, and the practicalities of implementation. The policy change that received the most favorable rating was adopting language that did not mention weight in public health messages. This was seen as a very low cost action with a high level of public acceptability and political feasibility. The shift from a weight-normative to a weight-inclusive approach also emerged as a public preference in a recent Canadian report where members of the community were engaged in a discussion, in person and online, about feasible action to promote healthy weight in children. The most popular idea expressed online was to turn away from a weight-normative approach in health promoting efforts as many participants expressed concern with the language on weight and instead preferred a focus on healthy living [159]. As this is a policy change that can be implemented with relative ease, OReilly and Sixsmith highlighted it as a viable and recommended action for governments to reduce harm caused by weight stigma and weight preoccupation [158].

Other promising policy options in OReilly and Sixsmith's analysis were implementing antiweight bias training for health professionals and establishing research guidelines that ensure the inclusion of measures of possible third-factor contributions to obesity research, such as socioeconomic status, physical activity, and dietary factors [158]. Several interventions to reduce weight bias among preservice and practicing health professionals have already been reported in the scientific literature with promising results [157, 160, 161]. In one study, over three hundred public health promoters were offered a single-day workshop on weight bias and related issues which led to significant decreases in antifat prejudice, decreased internalization of media stereotypes on weight and shape, and increased self-efficacy for addressing weight bias after intervention [157].

## 4. Summary of the Competing Approaches

The research demonstrates that a focus on weight is associated with adverse physical health and psychological well-being for patients and community members. Dieting is inextricably linked to significant physiological barriers to overall physical health that likely could have been prevented. The strain of unsuccessful weight loss attempts on physical health is not consistent with a beneficent and nonmaleficent approach to clinical practice and public health. Moreover, the weight-normative approach blames the individual rather than the process when weight loss attempts fail, which is then tied to body blame, body shame, internalized weight stigma, and decreased psychological well-being. Under the weight-normative approach, weight stigma likely filters into health care professionals' relationships with their patients, even if it is unintentional.

The weight-inclusive approach supports the health of people across the weight continuum and challenges weight stigma. Data from randomized controlled trials have upheld the efficacy of programs with a weight-inclusive emphasis, such as HAES. Specifically, participants following the HAES model achieved statistically and clinically significant improvements in physiological measures (e.g., blood pressure), behavioral practices (e.g., increased physical activity, decreased binge eating), and psychological measures (e.g., increased self-esteem, decreased depressive symptoms) and did not demonstrate any adverse outcomes, despite the fact that weight remained relatively unchanged. Other research has supported the weight-inclusive approach, such that living in a body-accepting environment (i.e., one without weight stigma) is associated with higher body appreciation and lower habitual appearance monitoring, independent of BMI. The weight-inclusive approach, then, upholds the ethical principles of beneficience and nonmaleficience and can be used as a springboard for generating additional clinical and public health interventions. Points of intervention, based on targeting the variables that are connected to reduced physical health and well-being (see Figure 2), as well as the mechanisms of action between the variables, are offered for health professionals who work with patients or within public health settings.

Returning to the vignette in the Introduction, we now frame the health care encounter between the doctor and patient through the lens of weight-inclusion and well-being instead of the pursuit of weight loss, taking into account how little time doctors get to spend with patients during a typical office visit.

Jasmine is waiting in the exam room and her chart shows that her weight today is up five pounds from her last visit two years ago, putting her BMI at 32. Her blood pressure was borderline high in contrast to the normal readings in previous visits. Although Jasmine's labs were normal in past visits, they are out of date. When Dr. Johnson greets her today, Jasmine seems anxious and tells Dr. Johnson, “I almost did not come in today knowing my weight is up from the last time I was here and you suggested a diet. I feel like such a failure. However, I need help for my migraines, so here I am.” Dr. Johnson and Jasmine look at each other, there is a beat of silence, and they both sigh.

Dr. Johnson says, “You know, Jasmine, I have been reading the research on weight loss interventions and weight-cycling and I'm realizing that if the same thing happens to almost everyone, it probably is not the fault of the person, it is probably more about the process itself. So, instead of focusing on weight loss, I'm encouraging my patients to think about what makes them feel better in their everyday lives; emotionally and physically. For example, do you feel better when you eat more fruits and vegetables, drink more water, take a walk with a friend, meditate to relieve stress, and get enough sleep? There's good evidence that those behaviors are going to make you healthier and feel better even if your weight does not change.”

Jasmine is a bit surprised by Dr. Johnson's shift and says, “Well, typically, when my weight loss slows down or stops completely, I stop doing any of those things you mentioned that would help me feel better and be healthier.” Dr. Johnson says, “I understand, but we're going to turn the focus from your weight to your health. Because those behaviors are linked to health, why not do them anyway?”

Jasmine smiles at Dr. Johnson and says, “It sure would be easier to come back and see you the next time I'm supposed to if I did not have to lose weight first.”

Dr. Johnson replies, “I do not want anything to stand in the way of you getting your medical care, including worrying that I might scold you. Now that we have a better plan, I am going to have the nurse retake your blood pressure.” Jasmine and Dr. Johnson then discuss treatment options for Jasmine's migraines.

Right before Dr. Johnson leaves the room, Jasmine shares one more quick concern, “I like the shift from weight to health, but there is this Weight Focusers group at work. If I do not go, I'll get charged a higher premium for my health insurance.”

Dr. Johnson says, “Let me know if I can help with that. The Affordable Care Act is supposed to allow you to follow your doctor's recommendation, and I have no evidence that Weight Focusers is going to make you healthier and lots of evidence that says that weight cycling is linked to poorer health.”

Jasmine leaves the doctor's office feeling hopeful and understood.

As Dr. Johnson finishes the chart note, she realizes that her own body is relaxed, her jaw unclenched. She feels like she has made a better connection with Jasmine and developed a sustainable treatment plan she can follow. Dr. Johnson is curious and maybe even a little eager to see what happens next. However, she does wonder what will happen if the reviewers do not see weight loss in this patient, or a goal of weight loss in the treatment plan.

## 5. Directions for Future Research

More research on the weight-inclusive versus weight-normative approach is sorely needed as many important questions remain unanswered. Research into the variety of expressions of weight stigma can reveal nuanced associations that advance scholars' understanding of its influence and expression. For example, weight stigma could be operationalized as weight-related teasing, bullying, discrimination, commentary, and objectification, and the source could also be operationalized (e.g., partners, health care system, family, friends, etc.). Similarly, decreased physical health and psychological well-being can be defined in many different ways and these operationalizations may reveal different relationships with weight and body-based variables.

Another alternative conceptualization would be to explore what happens in the absence of weight stigma, which would directly examine weight-inclusive approach. Those who do not experience weight stigma (whether because of their weight or their environment/community/culture) may demonstrate body appreciation and superior health and well-being. Although some research into positive body-accepting environments has begun [148, 151], these studies are in their infancy and would benefit from additional research. In addition, it would be useful to know whether individuals who transition out of weight-stigmatizing environments (e.g., away from stigmatizing partners or family members) receive health and well-being related benefits (and the extent of these benefits) or whether memories of being stigmatized continue to influence their health and well-being at a similar level. In the latter instance, perhaps mental health providers could work individually with patients to buffer internalized weight stigma and promote individual empowerment. In particular, interventions that emphasize self-compassion [161] may be useful for these therapeutic endeavors, as empirical evidence suggests that self-compassion is an adaptive mindset to cultivate in the context of improving body image and eating behavior [162–167]. Indeed, a 3-week online self-compassion intervention reduced body shame and improved body appreciation in community women; these women maintained these outcomes at a 3-month follow-up relative to a wait-list control group [150]. Among women high in dietary restraint, those who were induced to think self-compassionately after eating a doughnut as part of the experimental task (i.e., they were told that all people eat unhealthy foods at times and asked to not to be hard on themselves because “this little amount of food does not matter anyway”) were able to reduce their distress and disinhibited eating relative to a control group who did not receive the self-compassion induction [167].

Those working in patient settings and public health should investigate the impact of moving from a weight-normative approach to a weight-inclusive approach on their patients and communities. Researchers could explore the effects on patients' compliance and willingness to address health issues proactively when weight loss is removed from the equation. Qualitative designs could be used to garner rich data on the challenges and benefits of this change to health care, treatment adherence (e.g., more likely go to follow-up appointments for medical concerns), and overall health improvement. In addition, more research is needed to examine which particular components of the weight-inclusive approach, individually or in conjunction with other components, have the strongest connection to health improvement and promotion.

## 6. Conclusion

The weight-normative approach is not improving health for the majority of individuals across the entire weight continuum. Weight is overemphasized for higher-weight individuals (i.e., assumptions are made that they are unhealthy) and underemphasized for lower- or “average-” weight individuals (i.e., assumptions are made that they are healthy). Furthermore, we know that weight loss through dieting is not sustainable over time for the vast majority of higher-weight individuals and is linked to harmful consequences. Therefore, we argue that it is unethical to continue to prescribe weight loss to patients and communities as a pathway to health, knowing the associated outcomes—weight regain (if weight is even lost) and weight cycling—are connected to further stigmatization, poor health, and well-being. The data suggest that a different approach is needed to foster physical health and well-being within our patients and communities.

Advocates of a weight-inclusive approach assert that we are acting on behalf of our patients' and communities' interests when we centralize health for people at all points along the weight continuum and work to eradicate weight stigma in all settings, including health care and public health. This paper has reviewed the data in support of a weight-inclusive approach to foster physical and psychological well-being. We encourage both scholars and practitioners to study and document what happens when health professionals and their target populations shift their focus to developing sustainable healthy behaviors for every body.

## Figures and Tables

**Table 1:** Translating weight-inclusive principles into weight-inclusive practice.

Weight-inclusive principle	Weight-inclusive practice
(1) Eradicate weight stigma	Conduct trainings to inform other health care professionals about the weight-inclusive approach. Ensure medical offices have medical supplies and accommodations for all patients across the weight spectrum. Talk with patients' families, friends, and partners about the types of comments that are stigmatizing and negatively impacting the health of their loved ones. Promote the weight-inclusive approach and strategies for following it. *All health care professionals.

(2) Target internalized weight stigma	Help patients reduce placing blame on their bodies (and others' bodies). Challenge adoption of societal appearance ideals. Consider conducting cognitive dissonance interventions (e.g., [134]) to lessen adherence to unrealistic appearance ideals. *Mental health professionals

(3) Target body shame	Help lessen patients' embarrassment, hatred, and dissatisfaction toward their bodies by helping them define “beauty” more broadly and to appreciate their bodies. Cognitive dissonance interventions may help increase body appreciation. *Mental health professionals

(4) Redirect focus from external critique of weight and size to a “partnership” with the body	Direct attention to what is happening within their bodies rather than “picking apart” their appearance (e.g., lumps, appearance of moles, lack of energy, shortness of breath, etc.). This partnership with their bodies may help detect and prevent the progression of disease. *Physicians

(5) Look for signs of diminished well-being	Present options to alleviate distress and heighten life satisfaction; options should not be limited to medication. Know mental health professionals who follow a weight-inclusive approach in the community and refer patients as needed. *Physicians

(6) Look for signs of disordered, emotional, and/or binge eating	Rather than BMI, explore each patient's weight trajectory across time to detect unusual gains and losses that could be reflective of disordered eating. Do not praise weight loss. Do not immediately address weight gain with weight loss recommendations. *Physicians Explore with patients whether there is a connection between disordered eating patterns and emotional regulation. For instance, if they report bingeing behaviors, ask about how they felt at the time and contextual factors. If there is a connection, distress tolerance and mindfulness interventions (e.g., Acceptance and Commitment Therapy) may be helpful. *Mental health professionals

(7) Respond to requests for weight loss advice with a holistic approach	Respond (when asked by patients for advice or help with weight loss) with a holistic approach to health via encompassing and encouraging emotional, physical, nutritional, social, and spiritual health, rather than a weight-focus. *Physicians, nutritionists

(8) Sustain health promoting practices	Identify and facilitate access to healthy sustainable behaviors for patients. *All health care professionals

(9) Reconnect with food and internal cues	Help patients (a) abandon dichotomous thinking about foods as “good” and “bad” and the morality surrounding food restriction, (b) relearn how to recognize and respond to their hunger and satiety cues, and (c) determine how certain foods affect their bodies. *Nutritionists

*Health care professionals who may want to take the lead in implementing this principle within their practice. We encourage a team approach whereby physicians, mental health professionals, and nutritionists work together to ensure that a weight-inclusive approach is followed.
